# HPLC Analysis and Molecular Docking Study of *Myoporum serratum* Seeds Extract with Its Bioactivity against Pathogenic Microorganisms and Cancer Cell Lines

**DOI:** 10.3390/molecules28104041

**Published:** 2023-05-11

**Authors:** Abdullah Mashraqi, Yosra Modafer, Mohamed A. Al Abboud, Hanaa M. Salama, Emad Abada

**Affiliations:** 1 Biology Department, College of Science, Jazan University, Jazan 82817, Saudi Arabia; amashraqi@jazanu.edu.sa (A.M.); ymodafer@jazanu.edu.sa (Y.M.); malabboud@jazanu.edu.sa (M.A.A.A.); 2 Department of Chemistry, Faculty of Science, Port Said University, Port Said 42521, Egypt; hana_negm20010@yahoo.com

**Keywords:** bio-functional, *Myoporum serratum*, antimicrobial, anticancer, molecular docking

## Abstract

Natural constituents have been utilized to avoid humanity from various diseases, such as microbial infection and cancer, over several decades due to bioactive compounds. *Myoporum serratum* seeds extract (MSSE) was formulated via HPLC for flavonoid and phenolic analysis. Moreover, antimicrobial via well diffusion method, antioxidant via 2,2-diphenyl-1-picrylhydrazyl (DPPH) radical scavenging method, anticancer activities against HepG-2 cells (human hepatocellular cancer cell line), and MCF-7 cells (human breast cancer cell line), and molecular docking of the main detected flavonoid and phenolic compounds with the cancer cells were performed. The phenolic acids, including cinnamic acid (12.75 µg/mL), salicylic acid (7.14 µg/mL), and ferulic (0.97 µg/mL), while luteolin represents the main detected flavonoid with a concentration of 10.74 µg/mL, followed by apegenin 8.87 µg/mL were identified in MSSE. *Staphylococcus aureus*, *Bacillus subtilis*, *Proteus vulgaris*, and *Candida albicans* were inhibited by MSSE with 24.33, 26.33, 20.67, and 18.33 mm of inhibition zone, respectively. MSSE exhibited a low inhibition zone of 12.67 mm against *Escherichia coli* while showing no inhibitory activity against *Aspergillus fumigatus*. The values of MIC ranged from 26.58 to 136.33 µg/mL for all tested microorganisms. MBC/MIC index and cidal properties were attributed to MSSE for all tested microorganisms except *E. coli*. MSSE demonstrated anti-biofilm 81.25 and 50.45% of *S. aureus* and *E. coli*, respectively. IC_50_ of the antioxidant activity of MSSE was 120.11 µg/mL. HepG-2 and MCF-7 cell proliferation were inhibited with IC_50_ 140.77 ± 3.86 µg/mL and 184.04 µg/mL, respectively. Via Molecular docking study, luteolin and cinnamic acid have inhibitory action against HepG-2 and MCF-7 cells, supporting the tremendous anticancer of MSSE.

## 1. Introduction

Natural products are a valuable source for the discovery and development of pharmacological for the treatment and prevention of cancer as well as microbial infections. *Myoporum* is a small tree endemic to many nations and is a member of the Myoporaceae family. *Myoporum* species were typically used as horticultural plants and decorative windbreaks along roadsides. According to Dinda’s literature [1], George Foster discovered the genus *Myoporum* in 1786. According to Menut et al. [2] and Li et al. [3], *Myoporum* species are abundant in flavonoids, phenylethanoids, iridoids, terpenoids, and alkaloid contents. In addition to these secondary metabolites, most *Myoporum* species have secretory ducts that release a mixture of essential oils.

The few biological studies that have been done on *Myoporum* spp. in the past have concentrated mainly on *M. crassifolium*, *M. bontioides, M. insulare*, *M. montanum*, *M. acuminatum*, and *M. laetum*. It has been extensively studied *M. bontioides*, whose extract exhibits strong antifungal activity against *Fusarium oxysporum*, *Thielaviopsis paradoxa*, *Sphaceloma fawcettii*, *Colletotrichum musae*, *Mycosphaerella sentina*, *Alternaria alternata,* and *Pestalotia mangiferae* in addition to a repellent potential against *Plutella xylostella* [4,5] due to the existence of (−)-epingaione. The isolated sesquiterpene alkaloids (Myoporumine A and B), as well as dehydroepingaione of *M. bontioides,* also exhibited potent anti-methicillin resistant *Staphylococcus aureus* (anti-MRSA) effect with MIC values close to that of the standard vancomycin [6]. Strong antibacterial activity of aqueous extract of *M. montanum* leaves was documented against *Enterococcus faecalis*, *Moraxella catarrhalis*, and *Staphylococcus epidermidis*, due to the occurrence of (±)-myoporone, 11-hydroxymyoporone and 10,11-dehydromyoporone in the extract [7].

In New Zealand, *M. laetum* leaf juice has been utilized as an insecticide, and the outer bark has been used topically to treat skin eruptions and ulcers. For septic wounds, *M. laetum* leaf oils were used [1]. When *Myoporum* species were biologically evaluated, it was discovered that they have various biological effects, including anticancer, insecticidal, antibacterial, and anti-inflammatory properties [8,9,10]. Recently, The essential oil extracted from the leaf of *M. sandwicense* displayed excellent bacteriocidal and fungicidal potential toward *Streptococcus pyogenes* and *A. fumigatus*, respectively; also, essential oil of the wood visualized activity against *A. fumigatus*, *A. niger*, *M. gypseum,* and *S. pyogenes* [11].

In developed nations, cancer is now the second leading cause of death, and the clinical prognosis is still not excellent. Extensive research has been conducted over the past ten years to identify the mechanism by which oxidative stress could continue to cause cancer and chronic inflammation. According to earlier reports, proper dietary phytochemical intake may be able to lower cancer incidence [12,13,14,15]. The widespread utilization of some phytochemicals as therapeutic agents against different diseases is more significant [16,17,18]. Antiproliferative effect of 3,4′-dimethoxy-3′,5,7-trihydroxyflavone and 3,4′-dimethoxy-3′,5,7-trihydroxyflavone as the content of *M. bontioides* was reported against breast cancer cell line MCF-7 [8]. A molecular docking model was created to predict the most stable binding conformation of a ligand to a protein or enzyme and test for its quantitative structure-activity relationship (QSAR) model. It was created using a new scoring function that employs the Lamarckian genetics model to compute the change in free Gibbs energy and the inhibition constant.

Many biological activities, including antimicrobial activity, have been accurately predicted using this technique in silico [19]. The root-mean-square deviation RMSD value was used to assess the molecular docking model’s validity, and various scoring functions and docking programs were utilized to determine which ones were most important. Protein-ligand complexes for docking were used to classify some protein data bank identifications. Few studies have used the in silico docking model to explain the biological activities of natural products, although there are many different types of molecular docking models [20,21]. Many bioactive phytochemicals from plants have been studied over the past few decades, and some of them have impressive outcomes. It is only natural to wonder if we have already discovered the solution among all these natural substances for the greatest disease treatment. What part do compounds from plants play in lowering cancer cells and preventing recurrence in cancer patients as well as microbial infection [14,15,16,22]? This research provides some experiments on natural sources for repressing cancer and some microbial pathogens.

One of the most well-liked adjuvant therapies is phytotherapy, which is frequently the target of new infection drugs. The goal of plant-derived anticancer and antimicrobial therapeutics is to lessen side effects, improve chemotherapy sensitivity, and boost the treatment’s overall effectiveness [23]. Although there is a misconception that plants are harmless, they can actually have negative effects and even lessen the therapeutic benefits of conventional drugs. Consequently, it is essential to comprehend their chemical structure, pharmacological properties, proper dosage, and potential side effects if present. *Myoporum serratum* is uncommon for traditional medicinal applications in our country, therefore, the present study aimed to evaluate the antimicrobial, anti-biofilm, antioxidant, and anticancer activities of *M. serratum* seed extracts with molecular docking interaction of the main detected flavonoid and phenolic compounds with cancer cell line proteins.

## 2. Results and Discussion 

### 2.1. Phytochemical Constituents

**Figure 1:**
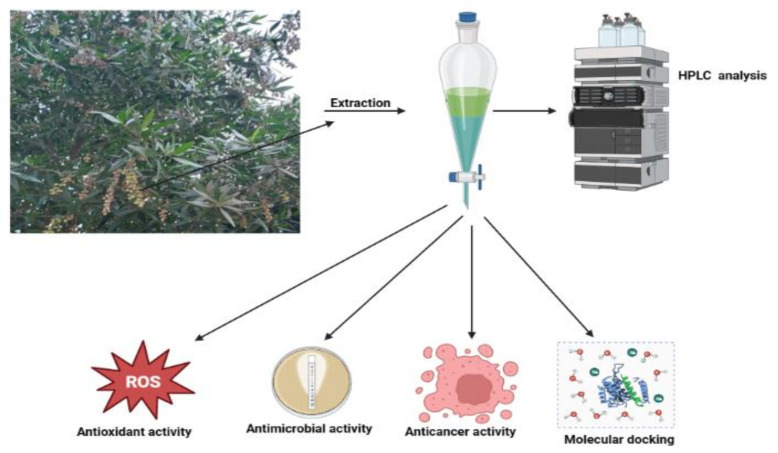
*Myoporum serratum* fruits subjected to the Phyto-constituents analysis by HPLC as well as other activities of the extract.

The collected fruits of *Myoporum serratum* seeds extract (MSSE) were prepared for phenolic and flavonoid characterization as well as many biological activities, including antimicrobial, antioxidant, anticancer activities, and molecular docking of the main detected flavonoid and phenolic compounds with the cancer cells (Figure 1).

**Figure 2:**
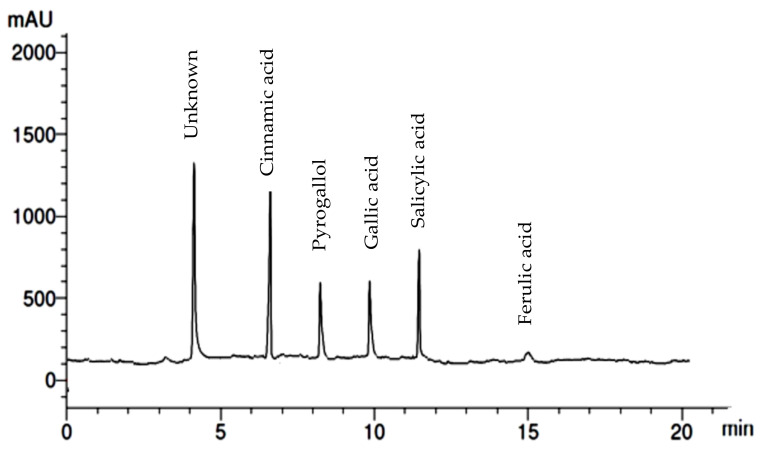
HPLC chromatogram of detected flavonoid compounds in *M. serratum* seeds extract.

**Figure 3:**
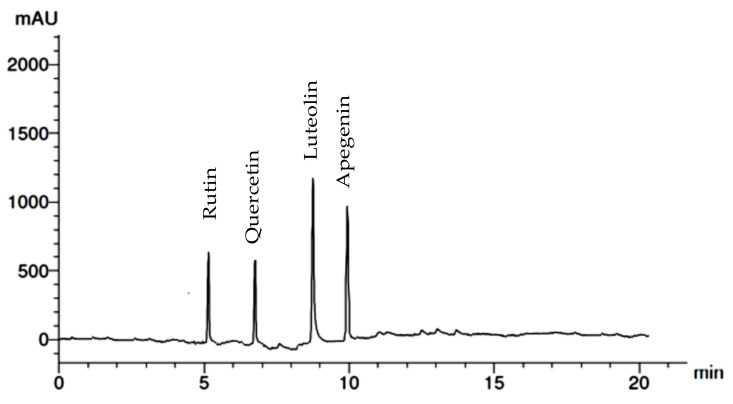
HPLC chromatogram of detected phenolic compounds in MSSE.

**Figure 4:**
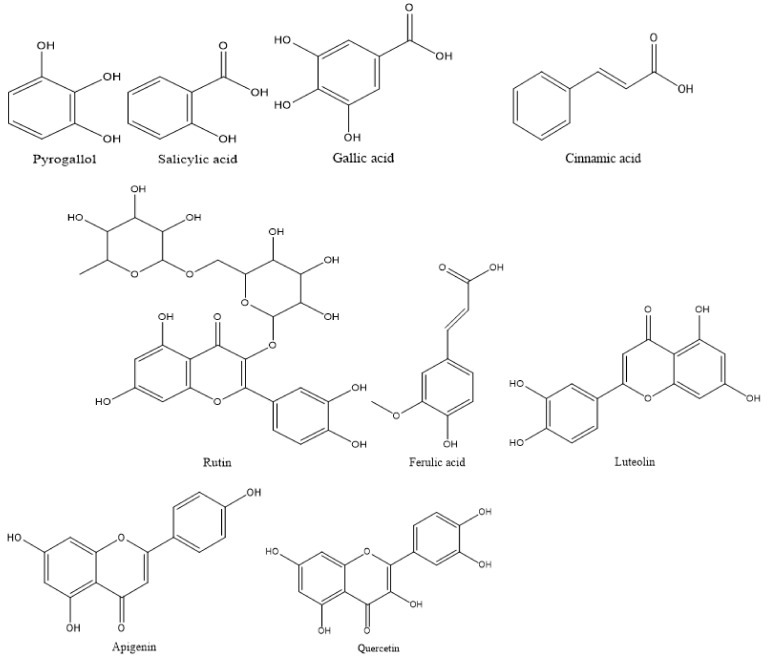
Structural composition of the detected phenolic and flavonoid compounds in MSSE.

Different phenolic and flavonoid compounds in the MSSE were recorded with varying times of retention and concentrations via HPLC analysis (Table 1 and Figure 2 and Figure 3). The detected compounds, either phenolic or flavonoid compounds, were different in the chemical constructions (Figure 4). High concentration was attributed to phenolic compounds cinnamic acid (12.75 µg/mL) followed by salicylic acid (7.14 µg/mL), while ferulic was detected in low concentration (0.97 µg/mL). HPLC analysis indicated that luteolin represents the main detected flavonoid with a concentration of 10.74 µg/mL, followed by apegenin at 8.87 µg/mL, besides the occurrence of quersestin and rutin but in moderate concentrations of 5.36 and 5.14 µg/mL, respectively. Numerous biological activities were documented in other reports utilizing these flavonoid and phenolic compounds. For example, antiangiogenic, antioxidant, and antitumorigenic effectiveness were attributed to cinnamic acid [24]. Su et al. [25] mentioned that cinnamic acid and its analogs, including ferulic acid, caffeic acid, isoferulic acid, and sinapic acid, display numerous therapeutic activities such as anticancer, anti-inflammation, and antioxidant activities in addition to immunomodulation. Moreover, melanoma cell proliferation was repressed using cinnamic acid [24]. Numerous therapeutic effects, including anti-inflammation, anticancer, and anti-allergy, were associated with luteolin; the anticancer mechanism of luteolin is linked to the stimulation of apoptosis, and prevention of cell propagation, metastasis, and angiogenesis [26]. Moreover, the antioxidant and antiperoxidative efficacy of luteolin were documented previously [27]. Yan et al. [28] investigated in vitro and in vivo the anticancer activity of apigenin. They found that apigenin suppresses multiple human cancers via numerous biological mechanisms, for instance, triggering cell apoptosis besides autophagy, encouraging cell cycle arrest, inhibiting cell migration and invasion, and enhancing an immune response. 

### 2.2. Antimicrobial Activity of M. serratum Seeds Extract

Results presented that the MSSE exhibited strong antibacterial activity against some tested bacteria, including *Staphylococcus aureus*, *Bacillus subtilis*, *Proteus vulgaris*, and *Candida albicans*, with inhibition zone values of 24.33, 26.33, 20.67, and 18.33 mm compared to inhibition zones 21.83, 25.33, 25.33, and 20.17 mm using standard antimicrobial agent, respectively (Table 2). In contrast, *Escherichia coli* was resistant to the extract with an inhibition zone of 12.67 mm compared with its sensitivity to gentamycin at 28.67 mm. Although the MSSE reflected inhibitory action against *C. albicans* as a unicellular fungus, it lacked efficacy against the filamentous fungus *Aspergillus fumigatus.* Some investigators documented a relationship between the chemical constituents of the plant extracts and antimicrobial activity [19,20,29]. In the current study, this correlation may be attributed to some phenolic and flavonoid compounds, such as luteolin, with noticeable antimicrobial activities against *S. aureus,* as mentioned in previous studies [30,31]. Who reported that bacterial DNA topoisomerase I and II were inhibited by luteolin leading to a decrement in the synthesis of nucleic acid and protein. A recent study also demonstrated the antibacterial activity against both *S. aureus* and methicillin-resistant *S. aureus* [32]. Zardi-Bergaoui et al. [33] reported the antibacterial activities of the essential oils extracted from the fresh and dry seeds of *M. insulare*. Different values of MIC of MSSE were recorded according to the tested microorganisms (Table 2). For instance, the recorded MIC was 73.33 ± 1.15 µg/mL, 42.08 ± 0.07 µg/mL, 135.33 ± 0.29 µg/mL, 26.58 ± 0.58 µg/mL, and 136.33 ± 0.07 µg/mL against *S. aureus*, *B. subtilis*, *E. coli*, *P. vulgaris,* and *C. albicans*, respectively. In this context, the MBC of the MSSE was also measured, which indicated that high MBC 545.33 ± 6.77 µg/mL was attributed to *E. coli*, followed by *C. albicans* (257.67 ± 5.77 µg/mL), *S. aureus* (133.33 ± 1.52 µg/mL), *P. vulgaris* (73.60 ± 0.17 µg/mL), and *B. subtilis* (73.33 ± 0.29 µg/mL). Excellent MIC of the MSSE was documented for some bacterial species, including *S. aureus*, *B. subtilis*, and *P. vulgaris*, while it was satisfactory for *E. coli* and *C. albicans*. These speculations were based on the scientific report of Kuete [34], who mentioned that the value of MIC is less than 100 µg/mL. The efficacy of the plant extract was considered good; if in the range of 100 µg/mL to 625 µg/mL, the plant extract was considered moderate, while the plant extract was considered weak if the MIC value was higher than 625 µg/mL.

Through the calculation of the MBC/MIC index, it is clear that the efficacy of the MSSE was cidal in the case of all the tested microorganisms except *E. coli*; it is static because the MBC/MIC index was more than four (Table 2). French [35] reported that if the MBC/MIC ratio is no more than four times the MIC, the natural compounds have cidal efficacy.

The biofilm of all tested microorganisms was influenced by the MSSE but with different percentages (Table 3). As well as the concentration of the extract MBC increased, The anti-biofilm increased. The biofilm of *S. aureus* was more affected by an inhibition of 81.25 ± 0.22%, followed by *B. subtilis* (70.71 ± 0.62%), *P. vulgaris* (72.17 ± 1.04%), *C. albicans* (57.33 ± 0.58%), and *E. coli* (50.45 ± 2.31%) at 75% of MBC. Robust inhibitory action of luteolin was observed on *S. aureus* and *Listeria monocytogenes* biofilm formation [31].

### 2.3. Antioxidant Activity of M. serratum Seeds Extract

**Figure 5:**
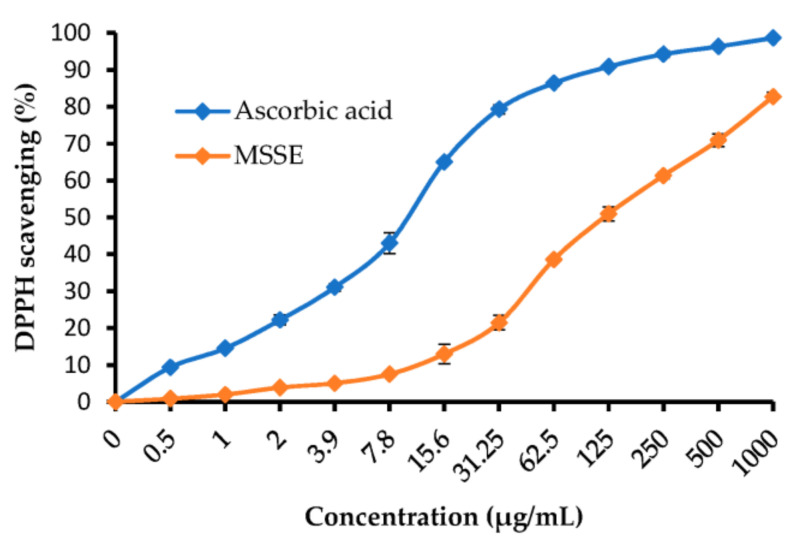
DPPH scavenging % of MSSE and ascorbic acid (3 replicates) at different concentrations. (3 replicates).

From the experiment of antioxidant activity, it is evident that the DPPH scavenging % increments with the rise of MSSE concentration (Table 4 and Figure 5). DPPH scavenging % was 50.97, 61.34, 70.95, and 82.74% at 125, 250, 500, and 1000 µg/mL of the MSSE, respectively. All outcomes were compared by using ascorbic acid as a standard antioxidant agent. The IC_50_ of the extract was 120.11 ± 3.69 µg/mL, while 10.21 ± 0.77 µg/mL was the IC_50_ of the ascorbic acid. Luteolin and apigenin were detected in the *Myoporum bontiodes* methanolic extract by Iwashina and Kokubugata [36]. In a recent study, the antioxidative efficacy of apigenin was documented by Kashyap et al. [37]. Moreover, they mentioned its application to overcome the problems associated with chronic diseases.

### 2.4. Anticancer Activity of M. serratum Seeds Extract

**Figure 6:**
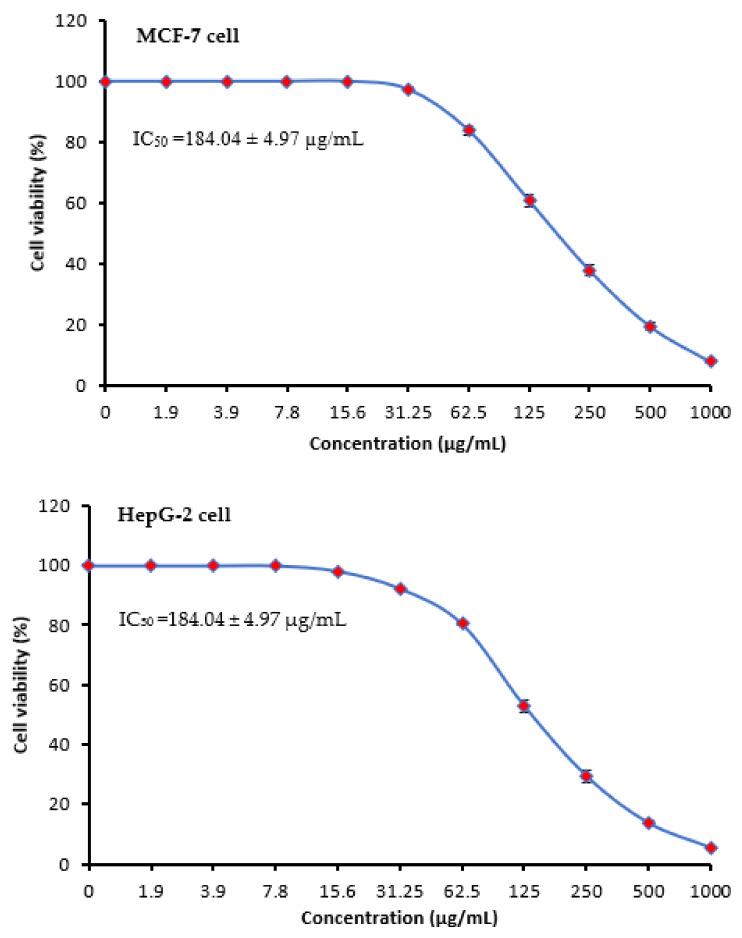
Cytotoxic activities of MSSE at different concentrations up to 1000 µg/mL (3 replicates) against MCF-7 and HepG-2 cells at 24 h exposure.

Cancer is the greatest hazardous illness that causes mortalities in all countries. Products of natural origin have afforded a rich source of therapeutic compounds comprising anticancer agents. Chemotherapy, hormone replacement therapy, radiotherapy, and surgery are all parts of the standard of care. There are frequent issues such as high recurrence and medication toxicity. As a result, the key to a higher level of success in the treatment of this disease is the combination of conventional treatment with a novel strategy using natural products [38]. Cytotoxic activity of MSSE against MCF-7 and HepG-2 cells was visualized in Table 5 and Figure 6. Cytotoxicity against the two cells increased with increasing the concentrations of MSSE in a dosage-dependent manner but with different levels of the inhibitory %. The anticancer potential against HepG-2 cells was observed at 15.6 µg/mL of the MSSE but not observed against MCF-7. At 250 µg/mL of the MSSE, the inhibitory % was 62.11 ± 1.73% and 70.57 ± 1.98%, while at 1000 µg/mL of the extract. The inhibitory % was 92.17 ± 0.42% and 94.59 ± 0.35% against MCF-7 and HepG-2 cells, respectively. IC_50_ was less than 140.77 ± 3.86 µg/mL against HepG-2 cells compared with IC_50_ (184.04 ± 4.97 µg/mL) against MCF-7. The anticancer activity may be due to the occurrence of cinnamic acid as the great content in the MSSE. The MSSE was tested against normal Vero cell line CCL-81 giving IC_50_ more than 345 µg/mL, and normal liver epithelial cell line (THLE-3) with IC_50_ 297.8 µg/mL (Data not tabulated). All results were compared to vinblastine sulfate as a positive control against HepG-2 cells with IC_50_ 2.93 ± 0.33 µg/mL (Table 5). The recent investigation reported that the moiety of cinnamic acid is ubiquitous in compounds of natural origin, and its derivatives display promising activity toward drug-susceptible and drug-resistant as well as multidrug-resistant cancers in vitro and in vitro [39]. The effect of natural cinnamic acid on breast cancer cells was documented [40] via induction of apoptosis. Numerous mechanisms have been suggested to explain the influence of natural compounds on the prevention and management of cancer. Some mechanisms against several cancer cells were reviewed [41], including disturbance of cell cycle regulation, autophagy mediation, apoptosis induction, cell migration, and invasion. The single morphology of MCF-7 cells in the microfluidic system exhibited that the extract of the Thymus had a significant influence on the membrane of cancer cells [42]. Apoptosis and autophagy were observed in HepG2 exposed to natural plant ferulic acid [43], which reported the appearance of alterations in cell morphology, nucleoli disruption, and the decrement of mitochondrial membrane potential. In another study, a block of the MCF-7 cell cycle at the G2/M phase was noticed as a result of exposure to natural plant products [44].

### 2.5. Molecular Docking

This research offers a virtual simulation of the interaction between the tested substance (the suggested drug) and the cell pathogen protein. Cinnamic acid and Luteolin, as the main detected compounds in MSSE, were subjected to this study’s protein-targeted inhibitors, which prevented the hormone notation of breast cancer cells (3HB5) and Hepatocellular Carcinoma (7JX9). This study tries to understand how each tested drug behaves against PDB proteins, including different orientations. The interaction parameters that were recovered for each docking molecule included the type of ligands, receptors, and interactions in addition to H-bond length, energy content, and scorning energy.

**Figure 7:**
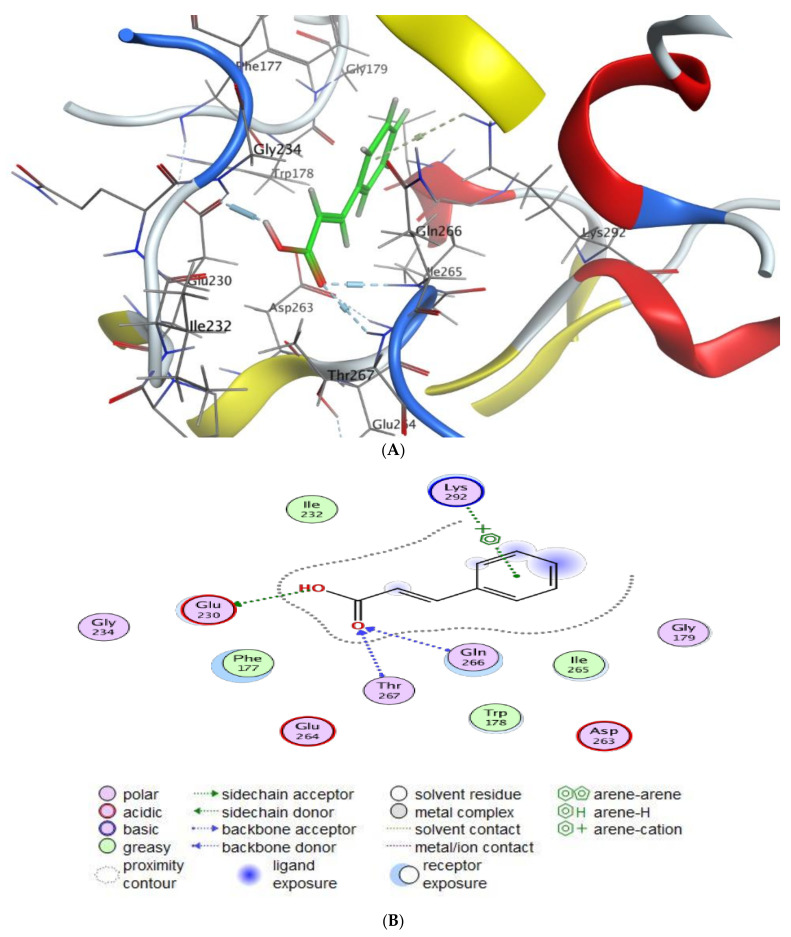

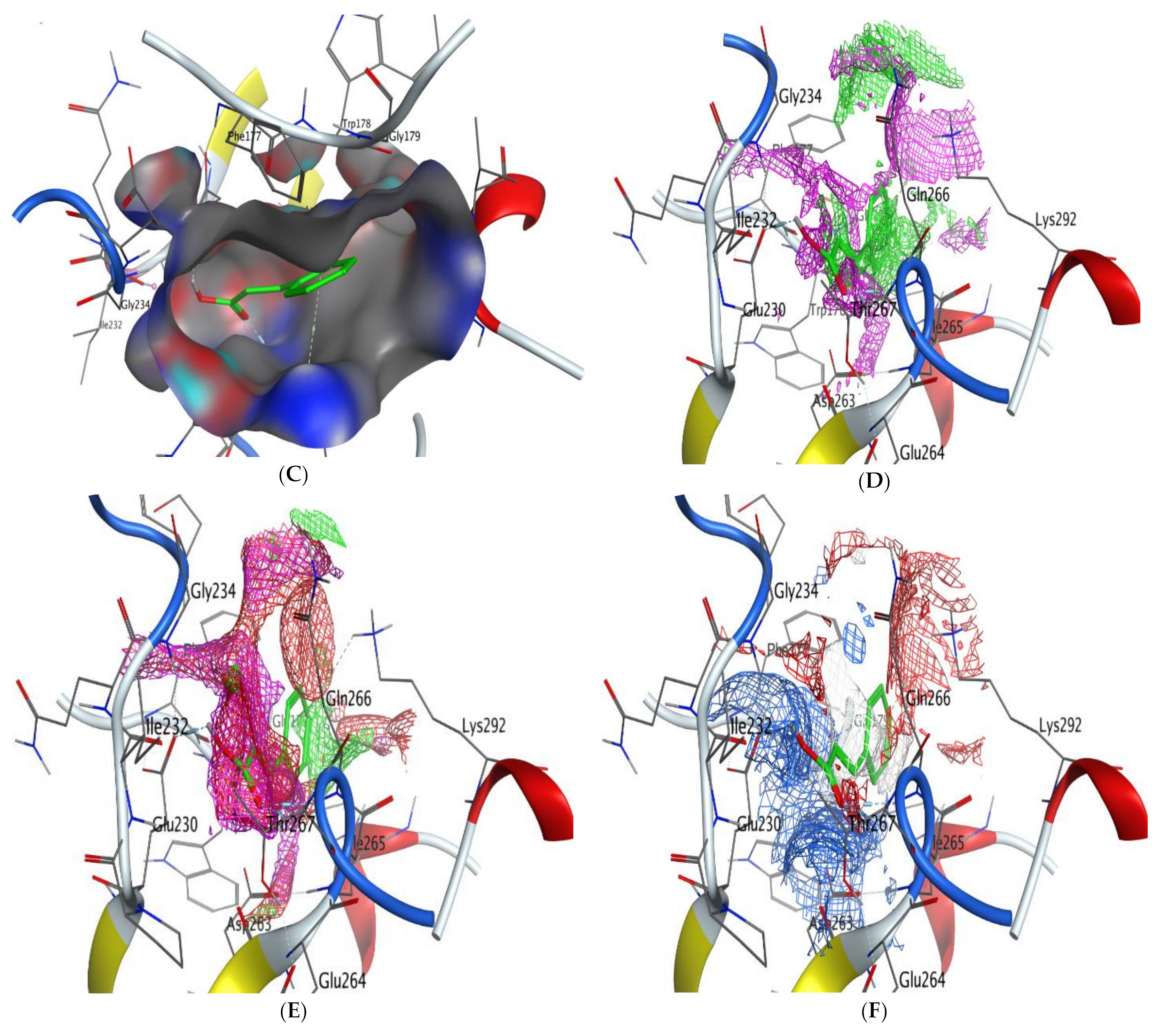
Molecular docking process of cinnamic acid with 7JX9. (**A**) The most likely binding conformation of cinnamic acid and the corresponding intermolecular interactions are identified, (**B**) The interaction between cinnamic acid and active sites of 7JX9 protein, (**C**) Molecular surface of cinnamic acid with 7JX9, (**D**) The contact preference of cinnamic acid with 7JX9, (**E**) Interaction potential of cinnamic acid with 7JX9, and (**F**) The electrostatic map of cinnamic acid with 7JX9.

**Figure 8:**
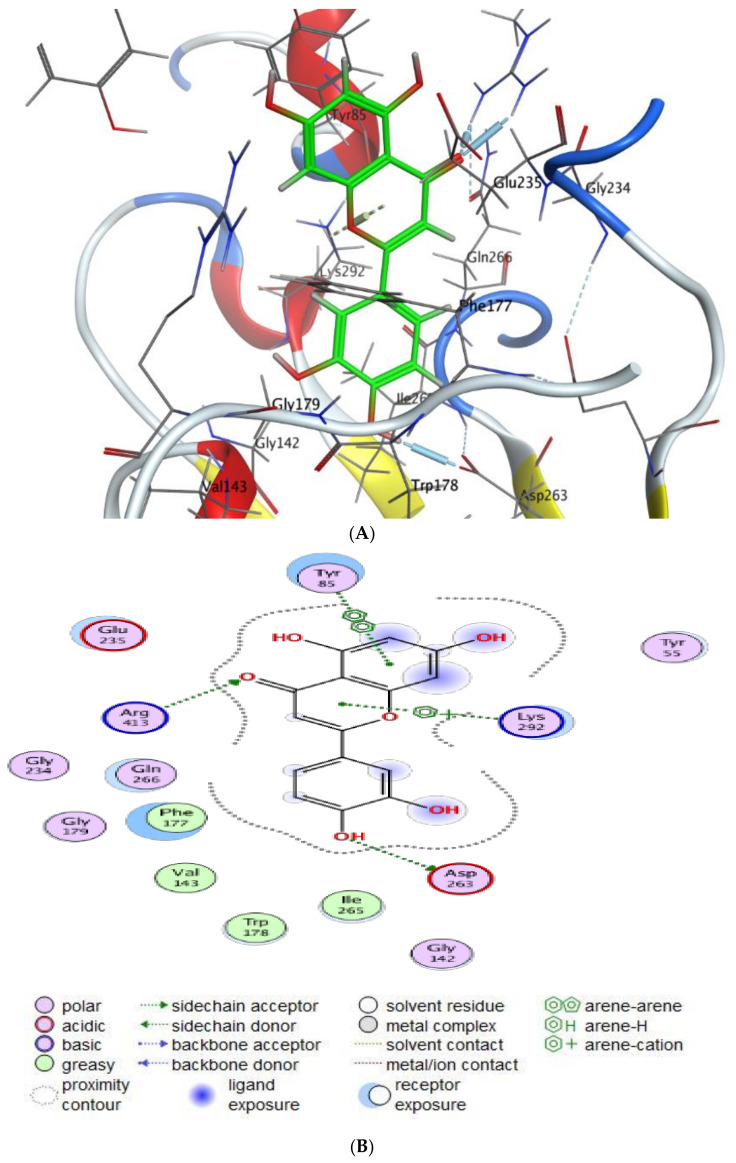

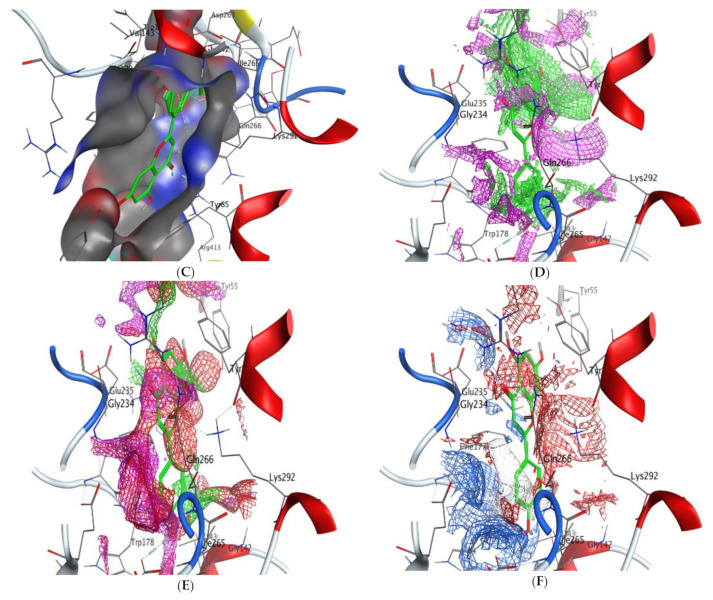
Molecular docking process of luteolin with 7JX9. (**A**) The most likely binding conformation of luteolin and the corresponding intermolecular interactions are identified, (**B**) The interaction between luteolin and active sites of 7JX9 protein, (**C**) Molecular surface of luteolin with 7JX9, (**D**) The contact preference of luteolin with 7JX9, (**E**) Interaction potential of luteolin with 7JX9, and (**F**) The electrostatic map of luteolin with 7JX9.

**Figure 9:**
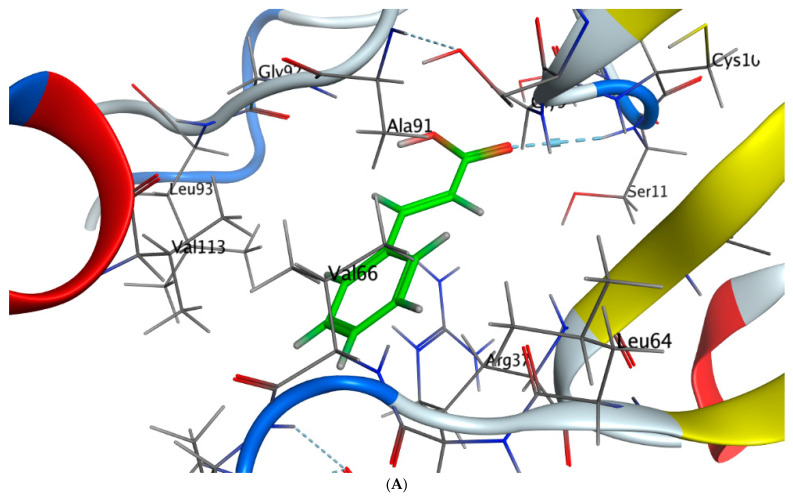

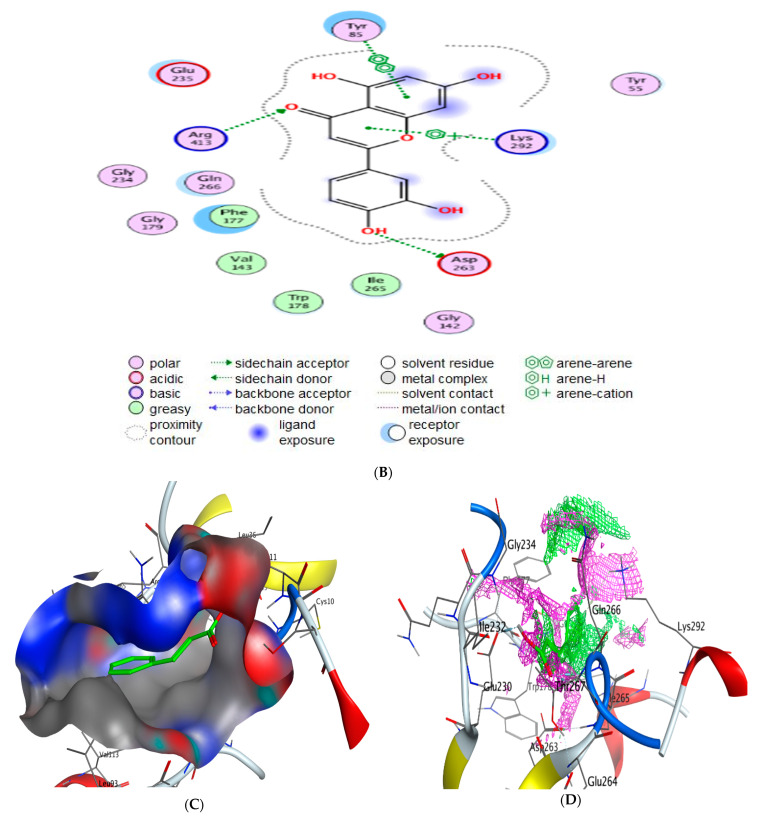

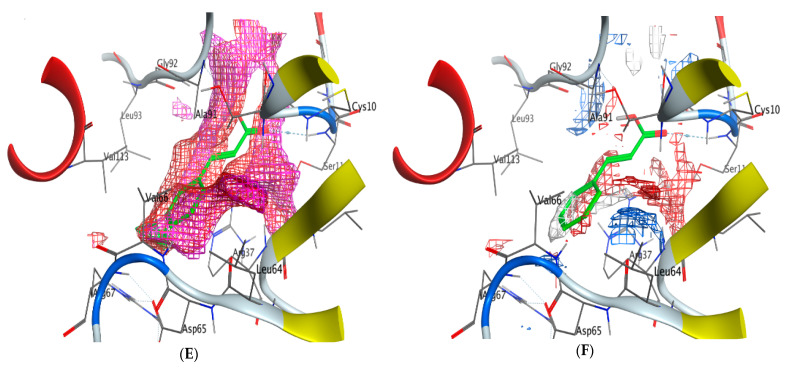
Molecular docking process of cinnamic acid with 3HB5. (**A**) The most likely binding conformation of cinnamic acid and the corresponding intermolecular interactions are identified, (**B**) The interaction between Cinnamic acid and active sites of 3HB5 protein, (**C**) Molecular surface of cinnamic acid with 3HB5, (**D**) The contact preference of cinnamic acid with 3HB5, (**E**) Interaction potential of cinnamic acid with 3HB5, and (**F**) The electrostatic map of cinnamic acid with 3HB5.

**Figure 10:**
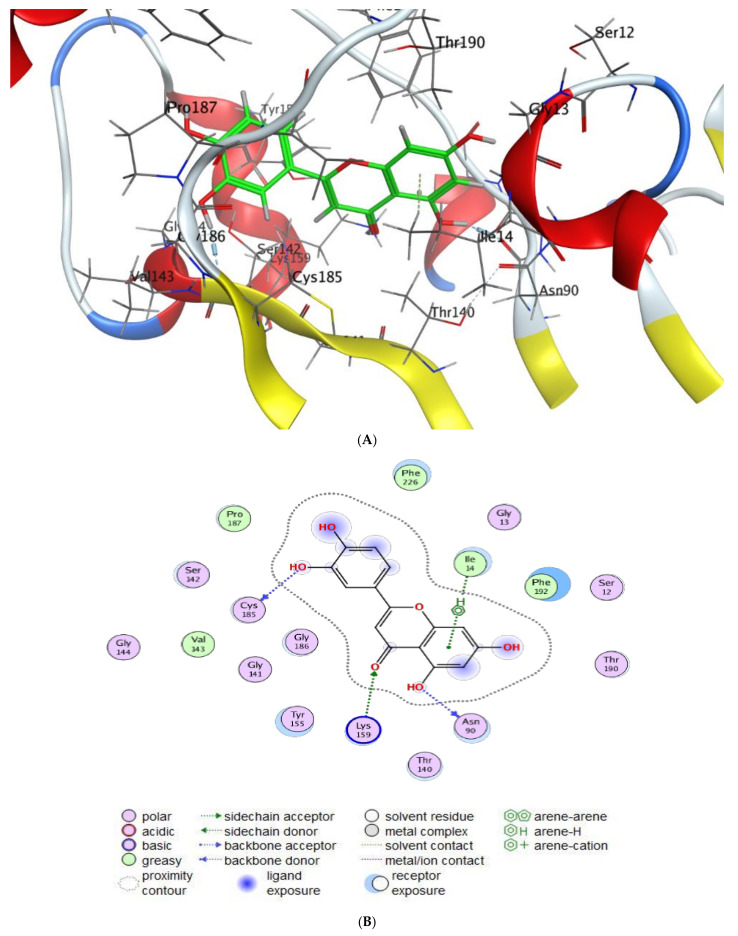

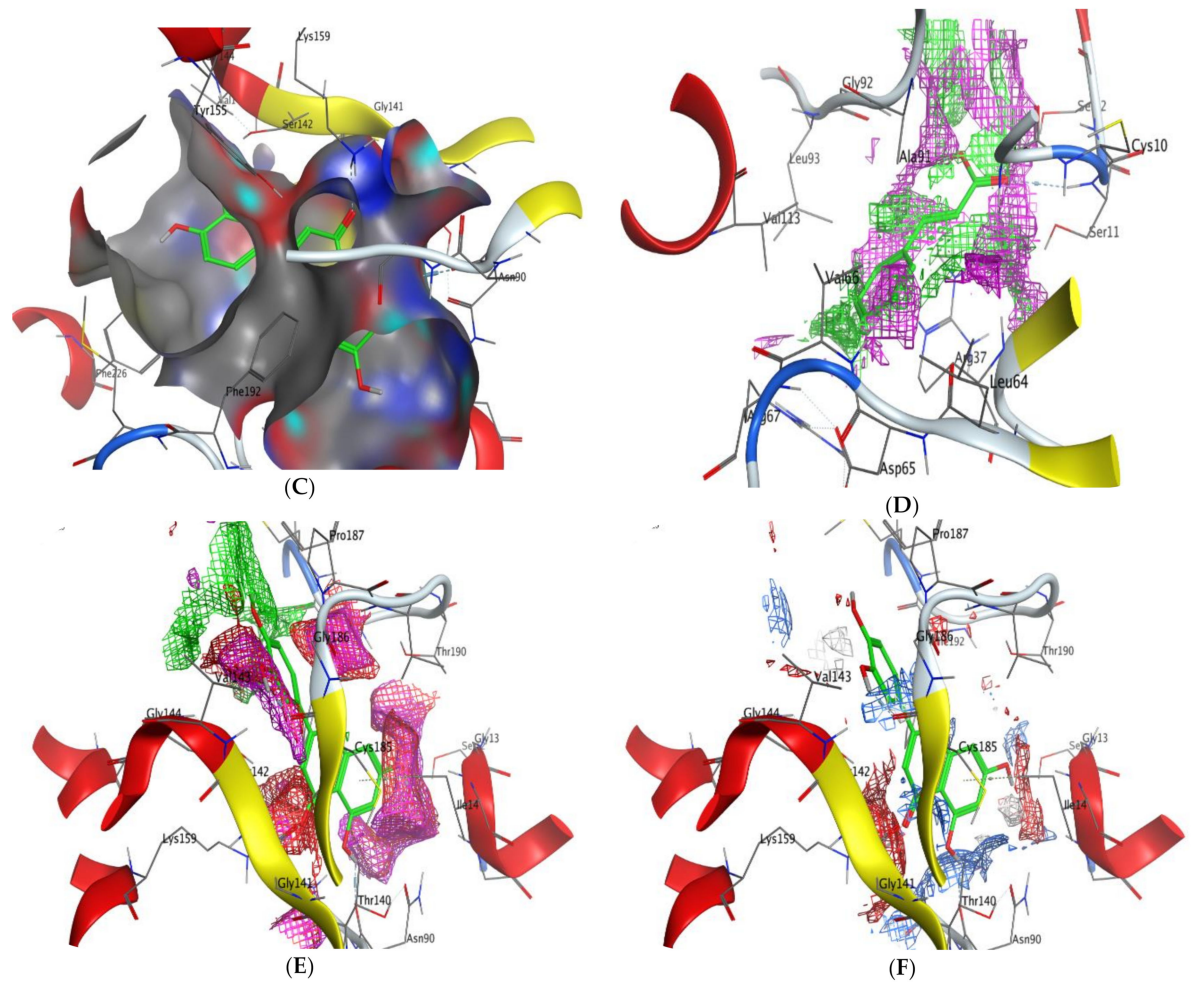
Molecular docking process of luteolin with 3HB5. (**A**) The most likely binding conformation of luteolin and the corresponding intermolecular interactions are identified, (**B**) The interaction between luteolin and active sites of 3HB5 protein, (**C**) Molecular surface of luteolin with 3HB5, (**D**) The contact preference of Luteolin with 3HB5, (**E**) Interaction potential of luteolin with 3HB5, and (**F**) The electrostatic map of luteolin with 3HB5.

The interaction patterns (Figure 7, Figure 8, Figure 9 and Figure 10) were exported together with basic docking parameters (Table 6, Table 7, Table 8 and Table 9).

Regarding tested substances, an apparent inhibitory effect was seen mainly for Luteolin, according to the following statements:

1. Scoring energy values that were effective and validated came from −6.48903 to −6.13434 kcal/mol^−1^ range with Hepatocellular Carcinoma (7JX9) active site and from −6.11443 to −5.9643 kcal/mol^−1^ range with breast cancer cells (3HB5) residue.
2. Numerous ligation sites for Luteolin interaction with (7JX9) are noted as O 28, O 27 6-ring through ASP 263, ARG 413, LYS 292, and TYR 85 amino acids receptors.
3. ASN 90, CYS 185, LYS 159, and ILE 14 were the contributing amino acids in the interaction of Luteolin with breast cancer cells (3HB5).
4. Cinnamic acid forms four hydrogen bonds with the Crystal Structure of Hepatocellular Carcinoma (7JX9) via GLU 230, GLN 266, THR 267, and LYS 292 to one acceptor hydrogen bond with breast cancer cells (3HB5) via the residue SER 11.
5. The tested compounds showed favorable RMSD values and interaction results.

Molecular Surface and Maps:

Molecular Surface and Maps is an integrated application for active site analysis. Create molecular surfaces, predict contact preferences, and calculate electrostatic maps. Color molecular surfaces by choosing from a variety of schemes such as temperature factor, pocket, lipophilicity, and electrostatic potential. Determine favorable locations of neutral, positive, and negative features in an active site. The electrostatic maps are calculated using the non-linear Poisson Boltzmann equation for receptor atoms and pseudo-ionic species. The advantage of the present method is that fully screened electrostatic potentials are used, and the domination of the potential by ionic groups is avoided. The red regions with negative molecular electrostatic potential are due to high electron density indicating a strong attraction between the protons, and the blue color corresponds to low electron density and weak interaction.

Antimicrobial and anticancer activities of several natural components were documented through molecular docking investigations [19,45]. Qanash et al. [21] confirmed the chlorogenic acid activity against *Proteus vulgaris* and human coronavirus (HCoV 229E). Al-Rajhi et al. [29] mentioned molecular docking study expected neophytadiene and luteolin inhibited *P. aeruginosa* and *E. coli*, respectively. According to Al-Rajhi et al. [15] *C. albicans* and *B. subtilis* proteins molecularly interacted with 2- benzenedicarboxylic acid and N-(4,6-dimethyl-2-pyrimidinyl)-4-(4-nitrobenzylideneamino) benzenesulfonamide to describe the inhibitory action of these compounds. Yahya et al. [19] also studied the molecular dicking interaction of *Aloe vera* gel loaded by chitosan nanoparticles loaded with the protein of *Helicobacter pylori*. In the present study, a high negative score of the free binding energy was observed, indicating the biological activity of naturally detected compounds in MSSE against cancer cells. Our findings about high negative scores were observed in other investigations using natural compounds [46,47].

Finally, although an explosion of knowledge regarding natural plant extracts over recent decades, we remain almost missed their mechanisms against numerous diseases. Therefore, it is necessary to study everything related to these plant extracts, including but not limited to their components and the toxicity of each compound separately, and to evaluate many of their activities with the use of more than one model of living organisms.

## 3. Materials and Methods

### 3.1. Reagents Used

Analytical grade chemicals were obtained from Sigma-Aldrich, Taufkirchen, Germany, including DPPH (2,20-diphenyl-1-picrylhy drazyl), Dimethyl sulfoxide (DMSO), ascorbic acid, trypan blue dye, crystal violet, L-glutamine, 25% Trypsin-EDTA, gentamycin, DMEM, RPMI-1640, Fetal Bovine serum, and bacterial growth medium. While Active Fine Chemicals Limited, Dhaka, Bangladesh, was the source of solvents and reagents.

### 3.2. Source of Cancer Cells

Mammalian cell lines: HepG-2 cells (human hepatocellular cancer cell line), MCF-7 cells (human breast cancer cell line), normal Vero cell line CCL-81 and normal liver epithelial cell line (THLE-3), were obtained from the American Type Culture Collection (ATCC, Rockville, MD, USA).

### 3.3. Tested Microorganisms

*Aspergillus fumigatus* (RCMB 002008), *Candida albicans* RCMB 005003 (1) ATCC 10231, *Staphylococcus aureus* ATCC 25923, *Bacillus subtilis* RCMB 015 (1) NRRL B-543, *Escherichia coli* ATCC 25922 and *Proteus vulgaris* RCMB 004 (1) ATCC 13315 were obtained from the Regional Center for Mycology and Biotechnology, Cairo, Egypt.

### 3.4. Plant Material Collection and Extraction

The plant material *Myoporum serratum* seeds were collected at the end of December 2022 from cultivated trees in Egypt. The seeds were washed with distilled water to remove dust, dried in the air, and dried in an oven at 50 °C for constant weight. The dried seeds were ground to fine powder, then extracted with methanol (100 g/500 mL) in a rotary evaporator, then the extract was concentrated and kept at 4 °C for analysis and further biological activities.

### 3.5. HPLC Analysis of the Extract Myoporum Serratum Seeds Extract (MSSE)

MSSE in five microliters was injected into HPLC (Agilent 1260 series, Agilent Technologies, Santa Clara, CA, USA) under the following brief conditions: the separation was performed on an Eclipse C18 column. In a 0.9 mL/min flow rate, water was used as the mobile phase along with 0.05% trifluoroacetic acid in acetonitrile and a column temperature of 40 °C. The UV detector was set to 280 nm to detect phenolics and flavonoids compared to injected standards [48].

### 3.6. Antimicrobial Activities of MSSE Using Agar Well Diffusion Method

The agar well diffusion method is the most important method used to estimate the antimicrobial activity of MSSE.

The whole dried agar surface is equally striped in three different directions. Allowing the agar surface to dry for not more than a quarter of an hour, a hole with a diameter of 6 to 8 mm is drilled aseptically, and a volume (100 µL) of the extract solution at the required concentration is injected into the well. After placing the extract solution, plates should be incubated within a quarter of an hour after they have been disposed of. After incubation times of two days (fungus used), the diameters of found inhibition zone (in mm) surrounding the wells must be measured to the closest entire millimeter. Gentamycin and ketoconazole were applied as positive control for inhibiting bacteria and fungi [49].

### 3.7. Broth Micro Dilution Method for MIC and MBC Detection

The reason for calling this method “microdilution” is that it requires fewer quantities of broth dispensed in antiseptic, plastic microdilution trays with circular or conical bottom wells. Every well has to include 0.1 mL of broth. The broth microdilution technique reported in M07 is the same methodology determined in ISO 20776-1.

Preparing and Storing Diluted MSSE

Intermediate twofold dilutions of MSSE volumetrically in 10 mL broth were made to prepare microdilution trays.

The dispensing device then delivers 0.1 (±0.02) mL into every 96 wells of a standard tray.

Inoculum Preparation and Inoculation

The inoculum will be prepared by making a direct broth or saline suspension of isolated colonies chosen from an 18 h agar plate. The suspension will be adjusted to obtain turbidity equivalent to a 0.5 McFarland standard. This step will produce a suspension of about 1 to 2 × 10^8^ colony-forming units (CFU)/mL for *Escherichia coli*. Within a quarter an hour after standardizing the inoculum, inoculate every well of a microdilution tray employing an inoculator device that provides a volume that does not override 10% of the volume in the well. Incubate microdilution trays for 16 to 20 h in an ambient atmosphere incubator within a quarter an hour of adding the inoculum. The least concentration of antimicrobial agent is the minimal inhibitory concentration (MIC), which entirely prevents the organism’s growth in the tubes or microdilution wells as discovered by the naked eye or measured OD at 600 nm [35].

### 3.8. Microtitre Plate Assay for Biofilm Quantification

By using 96-well polystyrene flat-bottom plates, it assessed the impacts of extracts on biofilm formalization. Fleetingly, 300 μL of inoculated caller trypticase soy yeast broth (TSY) (required concentration of about 10^6^ CFU/mL) was aliquoted into every well of microplate and cultured with sub-lethal concentrations (75, 50, and 25% of MBC) before performing. Controls were wells having medium and others not extracted and only with methanol. At room temperature, plates were incubated for two days. When the period of incubation ended, the supernatant was cleared away, and every well was entirely cleaned with antiseptic filtered water to dispose of free-floating cells; then, plates were air-dried for an hour, and the biofilm composed was colored for a quarter an hour at room temperature with 0.1% aqueous solution of crystal violet. The next step after incubation was to the excess stain by disposing of washing the plate three times with antiseptic filtered water. Finally, the dye bound to the cells was solubilized by adding 250 μL of 95% ethanol to every well. After a quarter an hour of incubation, absorbance was measured using a microplate reader at a wavelength of 570 nm [50].

Blank represented the absorbance of media only. The sample represented the absorbance of the test organism from treatment, while the control represented the absorbance of the test organism without any treatment. Biofilm inhibition ability of sample = (1 − (absorb. Sample − absorb. Blank))/(absorb. control − absorb. Blank)) ×100.

### 3.9. Antioxidant via DPPH Radical Scavenging Activity

DPPH radical was prepared in methanol (0.004%*w*/*v*) and maintained at 10 °C in the dark. A methanol solution for the test compound was designed. Three ml of DPPH solution were mixed with a 40 uL aliquot of methanol solution. Ascorbic acid was used as a reference chemical, and its absorbance and that of the DPPH radical without an antioxidant were also assessed. Three replicates of each determination were made, and the average was calculated. The DPPH radical’s percentage inhibition (PI) was calculated using the formula:

PI = [{(*A*C − *A*T)/*A*C} × 100]

where *A*C = absorbance of the control at t = 0 min and *A*T = absorbance of the sample +DPPH at t = 16 min.

Based on graphic depictions of the dose-response curve, the 50% inhibitory concentration (IC50), or the concentration needed to inhibit the DPPH radical by 50%, was calculated [15].

### 3.10. Evaluation of Cytotoxic Effects of MSSE

Cell line Propagation

The growth of cells was done By RPMI-1640 medium added to 50 µg/mL gentamycin and 10% inactivated fetal calf serum. The cells were kept at 37 °C in a damp ambiance with 5% CO_2_. They were sub-cultured from 2 to 3 times a week.

Cytotoxicity estimation employing viability assay.

To estimate antitumor assays, the tumor cell lines were hung up in a medium that contained a concentration 5 × 10^4^ cell/resource in Corning^®^ 96-well tissue culture plates, then incubated for a day. Eight concentrations for each compound were carried out by adding the tested compounds into 96-well plates (3 replicates). Six vehicle controls with 0.5% DMSO media were engaged for every 96 well plates as a control. After a full day of incubation, the MTT test was used to estimate the number of viable cells. The media was ejected from the 96 well plates and subrogated with 100 µL of fresh culture RPMI 1640 medium without phenol red, then 10 µL of the 12 mM MTT stock solution (5 mg) MTT in 1 mL of PBS) to very well together with the untreated controls. By adding 5% CO_2_, the 96 well plates were incubated at ambient temperature for 4 h. An 85 µL aliquot of the media was eliminated from the wells, which had been replaced by adding 50 µL of DMSO to each well, mixed carefully with the pipette, and incubated at ambient temperature for ten min. After that, the optical density was assessed at 590 nm with the microplate reader (Sunrise, TECAN, Inc., San Jose, CA, USA) to define the number of viable cells accurately.

Furthermore, the proportion of viability was calculated as [(ODt/ODc)] × 100%, where ODt is the mean optical density of wells treated with the tested sample. At the same time, ODc is the mean optical density of wells left untreated. A survival curve of each tumor cell line is derived by plotting the relationship between surviving cells and drug concentration. GraphPad Prism software (www.graphpad.com San Diego, CA, USA) was used to estimate IC_50_ for each conc based on graphic plots of dose-response curves [51].

### 3.11. Molecular Modeling Study

The docking process using the molecular operating environmental (MOE) program is interested in interaction features between tested compounds and functional-cell proteins. The MOE 2019 package for molecular docking examined cinnamic acid and Luteolin. The goal of the molecular docking was to reveal the binding interactions between the mentioned chemicals and the Crystal Structure of Hepatocellular Carcinoma (PDB:7JX9) and also the crystal structure of the breast cancer cell line (PDB:3HB5) as well.

Preparation of the chemicals under examination:

PerkinElmer ChemOffice Suite 2015 was used to create sketches of the examined compounds, which were subsequently prepared for docking using the MMFF94 force field; the native ligands were brought down to their lowest energy state. The final form was created after 3D protonation and the inversion process. The configured compounds were saved in MDB format, ready for the docking process.

Preparation of target receptors:

1-The Crystal Structure of Hepatocellular Carcinoma and Breast cancer cell line were downloaded from the protein data bank (http://www.rcsb.org/pdb accessed on 19 July 2022a) as a PDB file with access code PDB: 7JX9 and 3HB5, respectively.

2-Hydrogen atoms added over selected receptors after water removal.

3-Connecting the receptor types automatically, then fixing the potential energies. 

4-Site-finder was applied to the protein amino acid line helix.

5-After the dummies were modified to fit over protein alpha-sites, docking could initiate.

Docking of the investigated compounds to target receptor:

Although the docking simulation procedure took various times to cover all tested compounds, it always took an average of 30 postures. These poses were modified using the London dG scoring system, which was enhanced twice using triangle Matcher techniques. By scoring energy values, the binding rank of the investigated substances was distinguished. For docked complexes, additional features included ligand type, receptors, interaction type, H-bond length, and energy content. The overall determining factor for the extent of interaction validity was expanded to include the H-bond length (<3.5). Moreover, the surface maps and interaction patterns were retrieved to confirm comparison features.

### 3.12. Statically Analysis

The ±standard deviation (SD) and ±standard Error (SE) means of three replicates were recorded. GraphPad Prism^®^ (version 5.0) software was applied to calculate IC_50_ of the activity of DPPH radical scavenging

## 4. Conclusions

Several phenolic and flavonoid compounds were recognized in the MSSE, for instance, cinnamic acid, salicylic acid, ferulic, luteolin, and apegenin. Good antimicrobial activity and anti-biofilm against *S. aureus*, *B. subtilis*, *P. vulgaris,* and *C. albicans* were attributed to MSSE. Moreover, the antioxidant activity indicated that DPPH scavenging % increment with the increase of MSSE concentration recommended the potential use of MSSE as a natural food preservative. The anticancer potential of MSSE was reported against HepG-2 and MCF-7 cell proliferation, supporting its application in the medicinal field. The docking interactions of cinnamic acid and luteolin with the active site amino acid residues of breast cancer cells (3HB5) and Hepatocellular Carcinoma (7JX9) are evaluated. The results exhibited that both of them have potential inhibitory. The protein receptor 7jx9 has the lowest binding energy with the most acceptable rmsd_refine values. It possesses the highest possible affinity into the binding site of luteolin by forming a strong hydrogen bond which is assumed to be essential for the activity.

## Figures and Tables

**Table 1:** Flavonoid and phenolic compounds detected in *Myoporum serratum* seeds extract.

Phenolic			Flavonoid		
RT	Compound	Concentration µg/mL	RT	Compound	Concentration µg/mL
6.8	Cinnamic acid	12.75	5.2	Rutin	5.14
8.8	Pyrogallol	4.55	7	Quercetin	5.36
10	Gallic acid	3.42	9	Luteolin	10.74
12	Salicylic acid	7.14	10	Apegenin	8.87
15	Ferulic acid	0.97			

**Table 2:** Antimicrobial activity, MIC, and MBC of the MSSE against tested microorganisms.

Tested Microorganisms	Inhibition Zone (mm)		MIC of MSSE	MBC of MSSE	MBC/MIC Index MSSE
	MSSE	Control			
*S.aureus*	24.33 ± 0.58	21.83 ± 0.29	73.33 ± 1.15	133.33 ± 1.52	1.82
*B. subtilis*	26.33 ± 1.15	25.33 ± 0.58	42.08 ± 0.07	73.33 ± 0.29	1.74
*E. coli*	12.67 ± 2.08	28.67 ± 1.53	135.33 ± 0.29	545.33 ± 6.77	4.03
*P. vulgaris*	20.67 ± 1.15	25.33 ± 0.58	26.58 ± 0.58	73.60 ± 0.17	2.77
*C. albicans*	18.33 ± 0.58	20.17 ± 0.29	136.33 ± 0.07	257.67 ± 5.77	1.91
*A. fumigatus*	NA	17.50 ± 1.80	-	-	-

**Table 3:** Anti-biofilm % of the MSSE against tested microorganisms.

Treatment	Anti-Biofilm %				
	*S. aureus*	*B. subtilis*	*E. coli*	*P.vulgaris*	*C. albicans*
25% of MBC	62.40 ± 1.73	62.94 ± 1.15	31.65 ± 1.15	59.33 ± 1.15	44.00 ± 0.78
50% of MBC	63.67 ± 0.29	67.30 ± 0.58	46.83 ± 1.58	70.17 ± 0.29	47.00 ± 1.00
75% of MBC	81.25 ± 0.22	70.71 ± 0.62	50.45 ± 2.31	72.17 ± 1.04	57.33 ± 0.58

**Table 4:** DPPH scavenging % of *M. serratum* seeds extract and ascorbic acid.

Concentration (µg/mL)	MSSE		Ascorbic Acid	
	DPPH Scavenging %	±SD	DPPH Scavenging %	±SD
1000	82.74	1.28	98.65	0.10
500	70.95	1.71	96.34	0.65
250	61.34	0.92	94.25	0.92
125	50.97	1.89	90.89	0.17
62.5	38.56	0.78	86.42	0.71
31.25	21.43	1.95	79.35	1.22
15.6	12.96	2.62	65.05	0.21
7.8	7.48	0.46	43.01	2.87
3.9	5.03	0.31	31.04	1.04
2	3.87	0.25	22.18	1.37
1	1.95	0.11	14.48	0.62
0.5	0.84	0.18	9.34	0.46
0	0	0	0	0
IC_50_	120.11 ± 3.69 µg/mL		10.21 ± 0.77 µg/mL	

**Table 5:** Cytotoxic activities of MSSE against MCF-7 and HepG-2 cells.

Concentration (µg/mL)	MSSE				Vinblastine Sulfate	
	MCF-7		HepG-2 Cells		HepG-2 Cells	
	Viability %	Inhibitory %	Viability %	Inhibitory %	Viability %	Inhibitory %
1000	7.83	92.17 ± 0.42	5.41	94.59 ± 0.35	3.27	96.73 ± 1.48
500	19.47	80.53 ± 1.09	13.68	86.32 ± 0.74	5.89	94.11 ± 1.30
250	37.89	62.11 ± 1.73	29.43	70.57 ± 1.98	10.92	89.08 ± 1.25
125	60.84	39.16 ± 2.18	52.97	47.03 ± 2.11	14.36	85.64 ± 0.31
62.5	83.95	16.05 ± 1.39	80.46	19.54 ± 0.68	19.24	80.76 ± 0.48
31.25	97.41	2.59 ± 0.75	92.35	7.65 ± 0.41	26.85	73.15 ± 1.25
15.6	100	0	98.17	1.83 ± 0.28	34.19	65.81 ± 0.50
7.8	100	0	100	0	45.06	54.94 ± 1.33
3.9	100	0	100	0	54.28	45.72 ± 1.42
1.9	100	0	100	0	60.94	39.06 ± 0.45
0.0	100	0	100	0	100	0.0
IC_50_	184.04 ± 4.97 µg/mL		140.77 ± 3.86 µg/mL		2.93 ± 0.33 µg/mL	

**Table 6:** Docking scores and energies of cinnamic acid and luteolin with the Crystal Structure of Hepatocellular Carcinoma 7JX9.

Mol	rseq	mseq	S	rmsd_Refine	E_Conf	E_Place	E_Score1	E_Refine	E_Score2
Cinnamic acid	1	1	−5.25012	0.96225	−37.5672	−72.1759	−9.03118	−23.385	−5.25012
Cinnamic acid	1	1	−5.14903	2.133763	−36.5926	−64.9391	−9.42912	−24.0258	−5.14903
Cinnamic acid	1	1	−5.13294	0.851975	−39.5336	−60.0528	−9.17796	−24.4316	−5.13294
Cinnamic acid	1	1	−5.10296	1.69946	−37.5272	−63.3036	−9.82104	−24.0133	−5.10296
Cinnamic acid	1	1	−5.08605	0.644272	−37.956	−51.767	−10.2895	−20.4546	−5.08605
Luteolin	1	2	−6.48903	1.592102	−27.0053	−86.8895	−11.4305	−40.1925	−6.48903
Luteolin	1	2	−6.40791	1.900243	−27.0096	−90.0438	−11.7922	−33.0823	−6.40791
Luteolin	1	2	−6.25776	1.593486	−30.0341	−102.073	−12.1727	−37.6265	−6.25776
Luteolin	1	2	−6.23779	1.485299	−24.4523	−110.889	−11.7833	−38.4676	−6.23779
Luteolin	1	2	−6.13434	0.917851	−27.2434	−84.5921	−12.2857	−35.6893	−6.13434

**Table 7:** Interaction of cinnamic acid and luteolin with the Crystal Structure of Hepatocellular Carcinoma 7JX9.

Mol	Ligand	Receptor	Interaction	Distance	E (kcal/mol)
Cinnamic acid	O 17	OE1 GLU 230 (A)	H-donor	2.80	−4.1
	O 19	N GLN 266 (A)	H-acceptor	3.04	−2.0
	O 19	N THR 267 (A)	H-acceptor	3.30	−0.6
	6-ring	NZ LYS 292 (A)	Pi- Cation	4.65	−0.6
Luteolin	O 28	OD2 ASP 263 (A)	H-donor	3.10	−3.8
	O 27	NH1 ARG 413 (A)	H-acceptor	2.97	−4.8
	O 27	NH2 ARG 413 (A)	H-acceptor	3.02	−1.7
	6-ring	NZ LYS 292 (A)	Pi-Cation	3.97	−2.8
	6-ring	6-ring TYR 85 (A)	Pi-Pi	3.98	−0.0

**Table 8:** Docking scores and energies of Cinnamic acid and Luteolin with Crystal Structure of Breast cancer cell line 3HB5.

Mol	rseq	mseq	S	rmsd_Refine	E_Conf	E_Place	E_Score1	E_Refine	E_Score2
Cinnamic acid	1	1	−4.78831	0.554468	−38.1018	−53.3074	−8.50818	−12.759	−4.78831
Cinnamic acid	1	1	−4.67368	1.864181	−37.6401	−58.6463	−8.51607	−19.7046	−4.67368
Cinnamic acid	1	1	−4.56755	1.101168	−39.3437	−54.2494	−9.44964	−20.8978	−4.56755
Cinnamic acid	1	1	−4.54845	1.782263	−39.8343	−56.9743	−8.96893	−18.9359	−4.54845
Cinnamic acid	1	1	−4.53663	0.688159	−37.7271	−66.7538	−8.70658	−16.5558	−4.53663
Luteolin	1	2	−6.11443	4.067323	−26.4148	−79.6864	−12.3615	−35.4336	−6.11443
Luteolin	1	2	−6.05433	0.856852	−30.4067	−107.456	−13.9814	−31.4485	−6.05433
Luteolin	1	2	−6.04351	1.407485	−25.4523	−117.441	−12.3122	−36.4046	−6.04351
Luteolin	1	2	−5.96586	0.519985	−26.7221	−108.051	−13.2917	−30.9524	−5.96586
Luteolin	1	2	−5.9643	2.209804	−27.7025	−82.7211	−12.102	−33.9415	−5.9643

**Table 9:** Interaction of cinnamic acid and luteolin with Crystal Structure of Breast cancer cell line 3HB5.

Mol	Ligand	Receptor	Interaction	Distance	E (kcal/mol)
Cinnamic acid	O 19	N SER 11 (X)	H-acceptor	3.51	−0.8
Luteolin	O 23	O ASN 90 (X)	H-donor	2.81	−2.7
	O 30	O CYS 185 (X)	H-donor	2.90	−2.3
	O 27	NZ LYS 159 (X)	H-acceptor	3.18	−3.9
	6-ring	CD1 ILE 14 (X)	Pi-H	3.76	−0.7

## Data Availability

Not applicable.
